# Biomarkers for traumatic brain injury

**DOI:** 10.1007/s00415-018-8855-2

**Published:** 2018-04-10

**Authors:** Ronak Ved, Malik Zaben

**Affiliations:** Department of Neurosurgery, Institute of Psychological Medicine and Clinical Neurosciences, Cardiff University, Cardiff, CF14 4XW UK

Traumatic brain injury (TBI) poses a major public health problem. It is amongst the leading causes of mortality in young people in developed countries, and many survivors of TBI suffer from persistent disabilities. As a result, there remains an unmet clinical need for the development of more robust diagnostic and prognostic indicators of TBI. Axonal injury is a key driver of the ongoing pathological process following TBI, causing chronic neurological deficits and disability, and has been the focus of research in this area to date. A popular approach has been the investigation of body fluid (serum, CSF and saliva) biomarkers to assess axonal injury in the acute setting. Biomarkers can be any quantifiable product serving as a marker of physiological insult, and recent studies have highlighted several substances that appear both promising and clinically relevant. However, it is likely that eventually the optimal model for assessing axonal injury in TBI is likely to involve multi-faceted components, including multiple biomarkers and select advanced neuroimaging modalities. If successful, early and reliable identification of axonal injury post-TBI has the potential to enhance current care by increasing speed and accuracy of diagnosis, providing prognostic information, allowing patient stratification and efficient allocation of rehabilitation services, and providing better understanding if the underlying pathology, and discovery of potential therapeutic targets.

In this article, we review three studies investigating candidate serum biomarkers of TBI, with a focus on their potential relationship with clinical outcomes within both human and animal models.

## Increases of plasma levels of glial fibrillary acidic protein, tau, and amyloid b up to 90 days after traumatic brain injury

Bogoslovsky et al. conducted a study which aimed to identify the trajectory of three specific serum biomarkers—tau, glial fibrillary acid protein (GFAP), and amyloid beta (AB42)—from the acute–subacute stages after TBI, compared with controls.

Thirty-four patients were identified from a cohort of cases recruited to a clinical trial (COBRIT—the Citicoline Brain Injury Treatment Trial). Blood samples had already been collected from these patients at 24-h, and 30- and 90-day post-injury. Age-matched controls were identified from a combination of bespoke recruitment (*n* = 19) and a commercial source (*n* = 50). High-sensitivity ELISAs, wherein investigators were blinded as to assay origins, were conducted to quantify serum biomarker levels. These data were then integrated with analysis of admission injury severity, Marshall CT grade, duration of post-traumatic amnesia (PTA) and clinical outcomes, and Glasgow Outcome Score Extended (GOSE).

Plasma levels of GFAP, tau, and AB42 were elevated after TBI at all three time points compared to controls. Receiver operated characteristic (ROC) analysis revealed excellent discrimination between controls and TBI-cases at 24-h for GFAP and tau (Area under curve 0.94 and 0.90, respectively). Serum tau and AB42 remained markedly elevated up to 90-day post-TBI, whereas GFAP levels declined rapidly. Marshall CT grade and duration of PTA weakly correlated to tau level at 30-day post-TBI; however, there was no correlation between GFAP or AB42 and these markers of injury severity. There was a weak correlation between day 30 AB42 and GOSE.

*Comment* This study adds to existing literature detailing the kinetics of serum biomarker kinetics after TBI. Furthermore, serum GFAP levels reliably differentiated TBI patients from controls at 24-h post-injury. AB42 and tau increased more gradually post-injury, and their elevation is sustained for months post-TBI. Combined with the correlation between AB42 and clinical outcome, the study highlights the potential of these biomarkers to act as potential diagnostic or prognostic clinical tools in the assessment of TBI patients.

However, the study design may have been optimised to assess the utility of biomarkers as a diagnostic tool if a cohort of disease control patients had been included in addition to healthy controls. The study also identified its patient cohort from a group recruited to a discontinued randomised trial of citicoline in TBI. It is unclear how many of the TBI patients were exposed to the drug, which may influence cell membrane properties and thus the ability of biomarkers to leak into serum post-TBI. Furthermore, gender differences in responses to TBI have been reported in the literature, and in this study there was a significant discrepancy in gender proportions in the patient cohort (85% male) vs the control group (51% male). Future human studies should aim to control for these and other confounding factors, (e.g. polytrauma, pre-hospital substance misuse, inpatient prescription drugs) when investigating the diagnostic and prognostic validity of serum biomarkers of TBI.

Bogoslovsky T et al. (2017) Journal of Neurotrauma 34 (1):66–73.

## Correlation of mechanical impact responses and biomarker levels: a new model for biomarker evaluation in TBI

This paper reports a study investigating rat head kinematics during head impacts, providing data supporting the validity of an animal model of TBI. Levels of biomarkers were measured in both CSF and serum (GFAP, IL-6, tau and AB42) after controlled mechanical head injuries were administered to anaesthetized rats. The authors hypothesised that this panel of biomarkers correlate to measured mechanical head injury impacts and, therefore, may be helpful in determining TBI severity.

The study utilises the Marmarou impact model, described in detail in the study methods. In brief, a 450 g weight was released onto anaesthetized Sprauge–Dawley rats from 1.25, 1.75 and 2.25 m to simulate varying severity of TBI, respectively. Impact kinetics such as power and acceleration were recorded electronically, and post-impact time to self-right was measured as a marker of behavioural response to the injury. Biomarkers were collected from CSF and the serum 24-h post-injury.

Biomechanical and behavioural responses differed significantly between all impact groups versus controls, and between the 1.75- and 2.25-m impact groups (*p* < 0.05). There were statistically significant raised levels of NF-H, GFAP and IL-6 in 2.25-m group compared to controls, and all other impact groups. There was no difference in AB42 levels in any of the impact groups compared to controls. Pearson’s correlation analysis showed pNF-H and GFAP levels in CSF and serum had positive correlation with impact power and time to surface right (*p* < 0.01). No significant correlation was noted between post-injury IL-6 or AB42 and injury severity or time to surface right.

*Comments* TBI assessment and study in humans is often influenced by multiple confounding factors, e.g. polytrauma, varied mechanism of injury, and substance misuse. This study reports the biomechanical impact kinetics associated with one animal model of TBI, which holds the advantage of providing a method of controlling for many of the confounders associated with human studies of TBI. The authors also purport that levels of NF-H and GFAP had positive correlation with head kinematics and, therefore, that these two substances are potential biomarkers for TBI. The model used in this study is thus purported to hold a unique ability to aid in elucidating the relationship between biomarker levels and severity of the mechanical trauma to the brain.

However, the model utilised restricts biomarker analysis to a single time-point post-injury and, therefore, biomarker kinetic profiles cannot be assessed unless modifications to the methods for serum and CSF collection were made. Indeed, levels of AB42 are thought to rise at 3–5 days post-injury at the earliest, potentially explaining the absence of elevation of this biomarker in the present study. The influences of the inhaled anaesthetic upon blood–brain barrier function and ergo biomarker levels must also be acknowledged as a potential confounding factor. Revisiting the authors’ hypothesis, this work identifies that part of the panel of biomarkers described (GFAP and NF-H in particular) correlates with measured mechanical head injury impacts in this animal model of TBI and, therefore, they may have utility in determining TBI severity, if this relationship can be studied and verified in carefully controlled human studies of brain injury.

Li Y et al. (2015) J Neurol Sci. 359(1–2):280–6.

## Comparative assessment of the prognostic value of biomarkers in traumatic brain injury reveals an independent role for serum levels of neurofilament-light

Neurofilament-light (NF-L) is a neuron-specific protein, wherein it functions as a component of the axonal cytoskeleton. In this study, serum and CSF NF-L levels were measured after TBI in humans, alongside analysis of neuroradiological injury scores (primarily the Stockholm CT Severity score), initial injury severity, and clinical outcomes. A total of 182 patients suffering TBI admitted to a single neurointensive unit were included. Serum NF-L levels were acquired, together with S100B and neuron-specific enolase (NSE). CSF-NF-L was measured in a sub-cohort (*n* = 84) who underwent ventriculostomies as part of their TBI care. Outcome was assessed 6–12 months after injury using the Glasgow Outcome Score (1–5).

Both serum and CSF levels of NF-L demonstrated elevated during the first 15 days after trauma. There was a correlation between serum and CSF NF-L levels. There was no correlation between CSF or serum NF-L and MRI or CT imaging parameters of injury severity (Stockholm CT severity score and degree of midline shift on MRI). There was, however, a statistically significant correlation between NF-L levels and clinical outcome (*p* = 0.0056). Furthermore, in a multivariate analysis, when compared to the correlation of NSE and S100B with several measures of clinical outcomes, the authors concluded that a significantly better model predicting TBI severity was obtained when these biomarkers were combined (*p* = 0.006). These results indicate that both serum S100B and NF-L are potential predictors of TBI outcome. The investigators thus purport that this study demonstrates that serum NF-L correlates with TBI outcome, even if used in models combined with other biomarkers less specific to neurons, such as S100B. This suggests that NF-L may hold an independent contribution to the prediction of outcomes after TBI, perhaps by reflecting key pathophysiological processes post-injury. These processes may not be possible to monitor via conventional neuroradiological biomarkers. This may explain the lack of correlation between NF-L and neuroimaging severity scores identified in this study.

*Comments:* Whilst numerous biomarkers have been shown to relate to TBI, many of these are expressed in multiple tissues and, therefore, the presence of polytrauma may be a significant confounding factor in their utility as diagnostic or prognostic tools in clinical practice. There was a statistically significant correlation between serum NF-L and clinical outcome after TBI identified in the present study. It is argued that this neuronal protein may, therefore, be a more specific indicator of axonal injury than other biomarkers. This patient cohort primarily consisted of severe–moderate TBI patients, (92%) and there was significant variability in the time points at which neuroimaging was performed, and when CSF or serum was sampled for each patient; a more regimented sampling regime would have been be desirable to facilitate more controlled analysis and interpretation of data. Whilst the 3-week half-life outcome was also assessed at a single time-point (6–12 months) post-TBI, despite the fact that many patients continue to improve up to and beyond 12 months post-injury. More regular patient follow-up, and more robust measures of neurological function (neuropsychological/cognitive testing) would be desirable in future work assessing clinical outcomes after TBI in relation to biomarkers.

No correlation between NF-L and conventional CT or MRI imaging was identified in this study. This may reflect the fact that diffuse axonal injury is difficult to assess using conventional neuroimaging techniques. Future studies, aiming to elucidate potential relationships between biomarkers and neuroimaging after TBI may need to consider utilising novel advanced imaging techniques, such as diffusion tensor imaging and tractography, to validate the presence of axonal injury within patient cohorts.

Al-Nimer F et al. (2015) PLoS ONE 10(7):e0132177

